# Predictors of stillbirths in Bangladesh: evidence from the 2004–2014 nation-wide household surveys

**DOI:** 10.1080/16549716.2017.1410048

**Published:** 2017-12-20

**Authors:** Tanvir Abir, Kingsley E. Agho, Felix A. Ogbo, Garry J. Stevens, Andrew Page, Milton A. Hasnat, Michael J. Dibley, Camille Raynes-Greenow

**Affiliations:** ^a^ Translational Health Research Institute, School of Medicine, Western Sydney University, Penrith, Australia; ^b^ Humanitarian and Development Research Initiative (HADRI), School of Social Science and Psychology, Western Sydney University, Penrith, Australia; ^c^ School of Medicine and Public Health, Faculty of Health and Medicine, The University of Newcastle, Callaghan, Australia; ^d^ Sydney School of Public Health, Edward Ford Building (A27), University of Sydney, Sydney, Australia

**Keywords:** Bangladesh, infants, mortality, predictors, stillbirths, under-five

## Abstract

**Background**: Globally, stillbirth remains a significant public health issue, particularly in developing countries such as Bangladesh.

**Objective:** This study aimed to investigate the potential predictors of stillbirths in Bangladesh over a ten-year period.

**Methods**: The Bangladesh Demographic and Health Surveys data for the years 2004, 2007, 2011 and 2014 (n = 29,094) were used for the study to investigate the predictors of stillbirths. Stillbirth was examined against a set of community, socio-economic and child characteristics, using a multivariable logistic regression model that adjusted for cluster and sampling variability.

**Results**: The pooled rate of stillbirth in Bangladesh was 28 in 1000 births (95% CI: 22, 34). Stillbirth rates were higher in rural compared to urban areas in Bangladesh. Mothers who had a secondary or higher level of education (OR = 0.59, 95%CI: 0.43–0.82, P = 0.002) and those with primary education (OR = 0.66, 95%CI: 0.55–0.80, P < 0.001) were less likely to experience stillbirths compared to mothers with no education. Mothers with more than two children were significantly less likely to have stillbirths compared to mothers with one child. Those from poor households reported increased odds of stillbirth compared to those from rich households.

**Conclusion**: Our analysis indicated that no maternal education, primiparity and poor household were predictors of stillbirths in Bangladesh. A collaborative effort is needed to reduce stillbirth rates among these high-risk groups in Bangladesh, with the socio-economic and health-related Sustainable Development Goals providing a critical vehicle for the co-ordination of this work.

## Background

The Global Burden of Disease, Injuries and Risk Factors Study 2015 (GBD 2015) reported that the rate of stillbirth has fallen worldwide by 47% since 1990, and more quickly from the year 2000 [1]. Despite this decline, recent studies have reported that global estimates of stillbirth ranged from 2.1 million [1] to 2.6 million [2] in 2015, and approximately 98% of those fetal deaths occurred in developing countries [1,2]. Variation in global estimates of the stillbirth rate may be due to access to data sources and modelling strategy, as both studies used the standard definition for stillbirth (fetal death after 28 weeks’ gestation).

The United Nations reported that Bangladesh made a significant improvement in reducing under-5 mortality rate during the Millennium Development Goals (MDG) era (between 1990 and 2015) [3]. Despite this achievement, Bangladesh remains a major contributor to stillbirth rates in South Asia [2] with a reported stillbirth rate of 20 per 1000 live births in 2015 [1]. Stillbirths have an enormous impact on mothers, families, health care professionals and the community [4]. Previous studies have quantified the direct [5,6] and indirect [4] financial costs for parents after an experience of stillbirth, however, the psychological and social costs associated with stillbirth have been described as unquantifiable [7].

Based on the health burden associated with stillbirth, there is a renewed focus at the global level on ending preventable stillbirths by 2030 (Sustainable Development Goal, SDG-3.2) [8,9]. Similarly, the Lancet Series on ending preventable stillbirths highlighted the need for policy formulation and ongoing research, particularly improved data collection to support the implementation of evidence-based initiatives [8]. In the context of this global goal, country-specific evidence would be helpful in informing targeted interventions and policy decision-making to reduce stillbirth in Bangladesh.

In Bangladesh, information on risk factors for stillbirths is limited at the national level. Previous studies conducted in rural areas [10,11] and the inner city of Dhaka [12] found that a lack of maternal education, older maternal age (≥35 years), history of alcohol intake and drug abuse were associated with higher rates of stillbirth. The generalisability of these findings to the broader Bangladesh population may be limited, given differences in socio-economic status and geographical regions. The burden of stillbirths can vary within a country, with economically disadvantaged communities having higher rates compared to their economically well-off counterparts [13].

Using a reliable and population-based maternal and child health data source (Bangladesh Demographic and Health Survey, BDHS), we provide nationally representative information on the rate and predictors of stillbirth. Our study aimed to investigate the predictors of stillbirth in Bangladesh, using the BDHS datasets for the period (2004–2014).

## Methods

### Data sources

Datasets for the years 2004, 2007, 2011 and 2014 from the BDHS were pooled and used for the study. We pooled data across time to increase sample size and statistical power, consistent with previous studies [14–16]. The BDHS data were collected by the National Institute of Population Research and Training (NIPORT), with technical support from Measure DHS through the Inner City Fund (ICF) International. A weighted total sample of 29,094 pregnancies over 28 weeks’ gestation for women aged 15–49 years were included in the final analysis (2004: n = 6,395; 2007: n = 5,409; 2011: n = 9,021; and 2014: n = 8,269). The data were weighted to ensure the representativeness of the survey results at the national level.

In the 2011 and 2014 BDHS, a new administrative region called ‘Rangpur’ was created, and when Rangpur was removed from the overall data sets, a total weighted sample of 27,540 pregnancies over 28 weeks’ gestation for women aged 15–49 years was obtained (2004: n = 6,395; 2007: n = 5,409; 2011: n = 8,315; and 2014: n = 7,421). Data with Rangpur (general Bangladesh population) and without Rangpur were reported in this present study to ensure robustness of the analyses. The average response rate for the four surveys was 98%. A detailed description of the survey methodology, sampling procedure and questionnaires used for data collection is provided elsewhere [17].

### Outcome variable

The study outcome was stillbirth, defined as death of a fetus of more than or equal to 28 weeks’ gestation, consistent with previous studies [1,2,12]. The outcome was recorded as a binary variable in the datasets, coded as ‘1’ for stillbirth and ‘0’ for no stillbirth.

### Study factors

The study factors included community, socio-demographic and child factors. These were selected based on previously published studies and availability of data [10–12]. The community factors were place of residence (urban or rural) and geographical region, covering divisions in Bangladesh, namely: Barisal, Chittagong, Dhaka, Khulna, Rajshahi, Sylhet and Rangpur. Socio-demographic factors included number of children ever born, age of mother at the time of the interview, mother’s working status, mother’s marital status, mother’s body mass index (BMI), parents’ level of education, mother’s age at index childbirth, desire for pregnancy, mother’s access to the media (television, radio or newspaper). Child factors comprised gender of the child, previous multiple births, previous death of a sibling and combined birth rank and interval. Based on previous studies [18,19], we combined birth order and interval in the analysis because of the impact of birth order that may be mediated by the birth interval. Household wealth index was constructed by NIPORT and ICF International [17], using the principal components analysis by assigning weights to three household characteristics; namely: type of floor and wall; access to electricity; and six household assets; namely, possession of a radio, television, bicycle, motorcycle, car and fridge. The household wealth index was ranked across the four surveys, where household wealth index was divided into three categories. The bottom 40% of households were arbitrarily classified as poor households, the next 40% as the middle households and the top 20% as rich households [20]. Type of cooking fuels available to household members at the time of survey will be referred to as ‘household air pollution from solid fuel’. Household air pollution from solid fuel were categorised as solid fuels (coal/lignite, charcoal, wood, straw/shrubs/grass, agricultural crop, animal dung) and non-solid fuels (electricity, liquefied petroleum gas (LPG), natural gas, biogas, kerosene).

### Statistical analysis

Frequency tabulations were first conducted to describe the distributions of data by years of the survey, followed by calculation of the rate of stillbirths, unadjusted odds ratios (OR) and their 95% confidence interval (CI) of all potential predictors.

A three-stage model was performed for the multivariable logistic regression analyses by following a conceptual model that was employed by Chowdhury et al. [21]. In the first modelling stage, community and socio-economic determinants were examined, and only significant variables associated with the study outcome at 5% significance level were retained in model 1. In the second stage, the significant variables in *model 1* were added to child demographic factors. In the final stage, media factors and environmental factor were added to significant variables in model 2 to determine factors associated with stillbirth. All analyses were performed in Stata statistical software version 14 (Stata Corp., College Station, TX, USA) that adjusted for sampling weights, intra-cluster variability and sampling design to provide population-based estimates.

### Ethics

The study used existing survey datasets that are available online by application, with all identifier information removed. The surveys were approved by the Ethics Committee of the ICF International, USA and the National Research Ethics Committee of Bangladesh Medical Research Council (BMRC), Bangladesh. We obtained approval from Measure DHS to download and use the data for the study.

## Results

### Characteristics of the study population

The majority of mothers were from the Dhaka administrative region (32.2%), with the smallest group from the Barisal region (5.8%). Half of the mothers belonged to the youngest age group (15–24 years, 50.1%), with 8.8% aged 35–49 years. Mothers with no schooling and those with only primary education were almost equally represented (43.7% and 45.5%, respectively). Approximately 18 out of every 100 households were categorised as wealthy, and 42 out of every 100 households were categorised as poor households. Female and male children were almost equally distributed (Table 1).

**Table 1.** Characteristics of the study population in Bangladesh, 2004–2014 (n = 29,094).

	With Rangpur (a) (n = 29,094)			Without Rangpur (n = 27,540)		
**VARIABLE**	n	n*	%*	n	n*	%*
**COMMUNITY LEVEL FACTORS**						
**Year of survey**						
2004	6287	6395	22.0	6287	6395	23.2
2007	5473	5409	18.6	5473	5409	19.6
2011	8986	9021	31.0	7527	8316	30.2
2014	8069	8269	28.4	6714	7420	26.9
**Cluster type**						
Urban	8965	6423	22.1	8242	6212	22.6
Rural	19,850	22,670	77.9	17,759	21,328	77.4
**Region**						
Barisal	3313	1685	5.8	3313	1685	6.1
Chittagong	5876	6472	22.2	5876	6472	23.5
Dhaka	5406	9354	32.2	5406	9354	34.0
Khulna	3296	2605	9.0	3296	2605	9.5
Rajshahi	4124	4609	15.8	4124	4608	16.7
Sylhet	3986	2815	9.7	3986	2815	10.2
Rangpur	2814	1554	5.3			
**SOCIOECONOMIC DETERMINANTS**						
**Mother’s Age (years) (n = 29,087)**						
15–24	14,271	14,576	50.1	13,102	13,920	50.6
25–34	11,890	11,953	41.1	10,571	11,239	40.8
35–49	2634	2558	8.8	2308	2375	8.6
**Mother working status (n = 29,090)**						
Not working	23,132	23,095	79.4	20,648	21,719	78.9
Working	5679	5995	20.6	5351	5818	21.1
**Mother BMI (kg/m^2^) (n = 28,939)**						
≤18	6329	6360	21.9	5597	5967	21.7
19–25	18,595	19,064	65.5	16,905	18,090	65.7
25+	3724	3515	12.1	3350	3337	12.1
**Maternal marital status**						
Currently married	28,282	28,572	98.2	25,519	27,041	98.2
Formerly married	533	522	1.8	482	499	1.8
**Maternal highest level of education (n = 29,079)**						
No schooling	12,235	12,712	43.7	10,930	11,969	43.5
Primary	12,775	12,939	44.5	11,524	12,246	44.5
Secondary or more	3785	3428	11.8	3527	3309	12.0
**Paternal highest level of education (n = 29,077)**						
No schooling	13,898	14,440	49.6	12,413	13,588	49.3
Primary	9608	9697	33.3	8690	9178	33.3
Secondary or more	5291	4940	17.0	4880	4756	17.3
**Household Wealth Index**						
Rich	5763	5118	17.6	5123	4860	17.7
Middle	11,526	11,684	40.2	10,568	11,178	40.6
Poor	11,526	12,291	42.3	10,310	11,502	41.8
**CHILD DETERMINANTS**						
**Sex (n = 28,685)**						
Female	13,861	14,019	48.2	12,547	13,285	48.2
Male	14,538	14,666	50.4	13,137	13,899	50.5
**Birth rank and birth interval**						
2nd/3rd birth rank, more than 2 years interval	10,675	10,935	37.6	9776	10,455	38.0
1st birth rank	9948	9996	34.4	9164	9556	34.7
2nd/3rd birth rank, less than or equal to 2 years interval	1924	1907	6.6	1677	1777	6.5
4th birth rank, more than 2 years interval	5178	5200	17.9	4453	4787	17.4
4th birth rank, less than or equal to 2 years interval	1090	1056	3.6	931	965	3.5
**Previous Death of Sibling**						
No	28,067	28,352	97.5	25,318	26,832	97.4
Yes	748	742	2.6	683	708	2.6
**Number of children born (n = 29,011)**						
1	7990	7999	27.5	7401	7675	27.9
2	8732	8868	30.5	8012	8485	30.8
3	5278	5412	18.6	4727	5110	18.6
4+	6733	6732	23.1	5795	6196	22.5
**Number of children under-five years**						
1–2	17,873	18,113	62.3	16,467	17,365	63.1
3 or more	10,942	10,981	37.7	9534	10,175	37.0
**MEDIA FACTORS**						
**Watches television every week (n = 29,011)**						
Yes	16,123	16,080	55.3	14,775	15,421	56.0
No	12,688	13,011	44.7	11,224	12,116	44.0
**Listens to radio every week (n = 29,088)**						
Yes	5158	5385	18.5	5019	5305	19.3
No	23,650	23,703	81.5	20,975	22,229	80.7
**Reads newspaper (n = 29,075)**						
Yes	4501	4115	14.1	4054	3902	14.2
No	24,291	24,960	85.8	21,930	23,621	85.8
**ENVIRONMENTAL FACTOR**						
**Type of cooking fuel (n = 26,325)**						
Solid fuel	2995	2943	10.1	2644	2846	10.3
Non-solid fuel	23,140	23,382	80.4	20,871	22,018	80.0

^&^Weighted for the sampling probability; n^&^ weighted ‘n’

*percentage did not add up to 100% because of missing values.

(a) Overall Bangladesh population

### Rates and predictors of stillbirths

**Figure 1.**
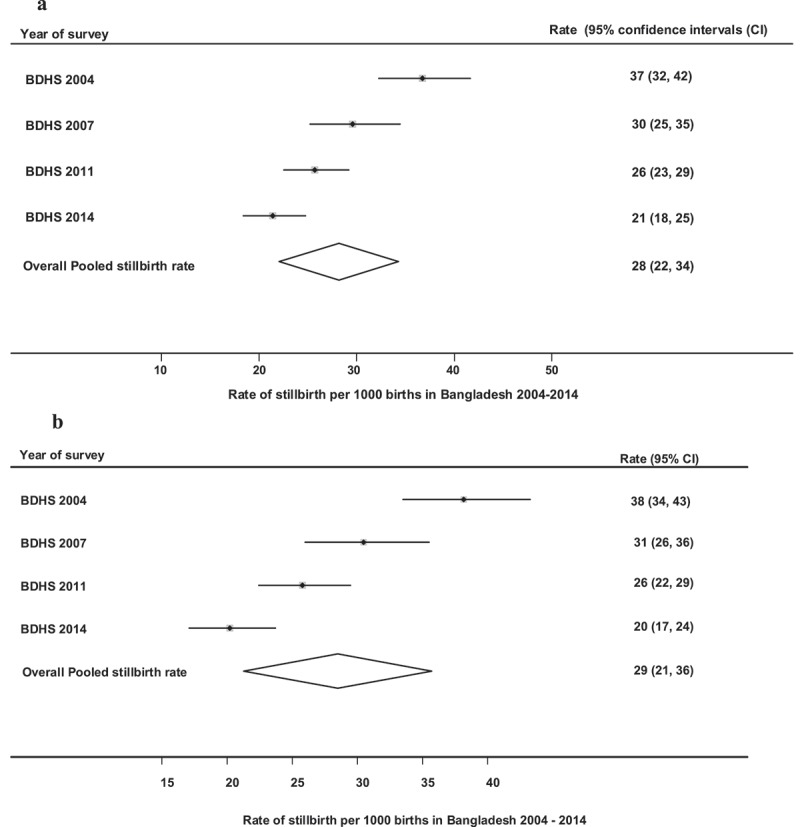
(a) Rate of stillbirth per 1000 births in Bangladesh (with Rangpur), 2004–2014. (b)Rate of stillbirth per 1000 births in Bangladesh (without Rangpur) 2004–2014.

As shown in Figure 1(a) (with Rangpur), the rate of stillbirth was 37 [95% confidence interval (CI): 32, 42] per 1000 births in 2004; 30 (95% CI: 25, 35) per 1000 births in 2007, 26 (95% CI: 23, 29) per 1000 births in 2011 and 21 (95% CI: 18, 25) per 1000 births in 2014. From 2004 to 2014, the overall rate of stillbirth was 28 (95% CI: 22, 34) per 1000 births. These results indicated that stillbirth decreased significantly in 2011 and 2014 compared to 2004, but in 2007 compared to 2011 and 2014, there was no significant decrease in stillbirth rate. In comparison to the population with Rangpur (Figure 1(a)), there was no significant differences in the rate of stillbirth in the population without Rangpur (Figure 1(b)).

The analysis showed that the rate of stillbirth was higher among rural mothers, older women, mothers with no schooling and mothers from poor households in Bangladesh (with Rangpur) [Table 2]. The stillbirth rate was significantly higher among households who reported non-solid fuel use and mothers who reported fourth birth order of child with more than 2 years’ birth interval.

**Table 2.** Rate and univariate analysis of stillbirth by study factors in Bangladesh, 2004–2014.

	With Rangpur (a)				Without Rangpur			
	Rate 95%[CI]	Unadjusted odds ratio			Rate 95%[CI]	Unadjusted odds ratio		
VARIABLE		OR	(95% CI)			OR	(95% CI)	
**COMMUNITY LEVEL FACTORS**								
**Cluster type**								
Urban	23 [19, 48]	1.00			22 [18, 26]	1.00		
Rural	30 [28, 43]	1.36	1.13	1.65	30 [27, 32]	1.39	1.14	1.69
**Region**								
Barisal	28 [19, 36]	1.00			28 [19, 36]	1.00		
Chittagong	26 [22, 30]	0.93	0.65	1.34	26 [22, 30]	0.93	0.65	1.35
Dhaka	27 [24, 30]	1.01	0.71	1.44	27 [24, 30]	1.02	0.71	1.45
Khulna	24 [18, 30]	0.87	0.57	1.32	24 [18, 30]	0.87	0.57	1.33
Rajshahi	33 [28, 39]	1.26	0.87	1.82	33 [28, 39]	1.26	0.87	1.83
Sylhet	33 [26, 39]	1.26	0.85	1.88	33 [26, 39]	1.27	0.85	1.90
Rangpur	36 [26, 46]	1.37	0.88	2.14				
**SOCIOECONOMIC DETERMINANTS**								
Mother’s Age (years)*								
15–24	28 [26, 31]	1.00			28 [25, 31]	1.00		
25–34	28 [25, 31]	0.99	0.85	1.15	28 [25, 31]	0.99	0.85	1.16
35–49	31 [24, 38]	1.09	0.85	1.40	31 [24, 38]	1.11	0.86	1.44
**Mother working status**								
Not working	35 [31, 39]	1.00			28 [26, 30]	1.00		
Working	33 [26, 40]	0.97	0.76	1.25	29 [25, 34]	1.05	0.88	1.25
Mother BMI (kg/m^2^)*								
≤18	30 [25, 34]	1.00			30 [25, 34]	1.00		
19–25	29 [26, 31]	0.97	0.82	1.15	28 [26, 31]	0.95	0.80	1.14
25+	24 [19, 29]	0.81	0.62	1.06	23 [18, 28]	0.78	0.59	1.03
**Maternal marital status**								
Currently married	28 [26, 30]	1.00			27 [25, 29]	1.00		
Formerly married	63 [41, 85]	2.28	1.56	3.32	59 [37, 81]	2.18	1.46	3.23
Maternal highest level of education*								
No schooling	34 [31, 38]	1.00			34 [30, 37]	1.00		
Primary	25 [22, 28]	0.72	0.62	0.84	25 [22, 28]	0.73	0.62	0.85
Secondary or more	20 [15, 25]	0.57	0.44	0.75	20 [15, 25]	0.59	0.45	0.77
Paternal highest level of education*								
No schooling	33 [30, 36]	1.00			32 [29, 35]	1.00		
Primary	28 [24, 31]	0.84	0.72	0.99	27 [24, 31]	0.85	0.72	0.99
Secondary or more	17 [13, 21]	0.51	0.40	0.65	17 [13, 20]	0.51	0.40	0.65
**Household Wealth Index**								
Rich	18 [14, 21]	1.00			17[14, 21]	1.00		
Middle	30 [26, 33]	1.71	1.35	2.17	29 [26, 33]	1.74	1.35	2.23
Poor	32 [29, 35]	1.85	1.46	2.35	31 [28, 35]	1.87	1.46	2.40
**CHILD DEMOGRAPHICS**								
Gender*								
Female	14 [12, 16]	1.00			15 [13, 17]	1.00		
Male	13 [12, 16]	0.97	0.79	1.19	14 [12, 17]	0.97	0.80	1.19
**Birth rank and birth interval**								
2nd/3rd birth rank, more than 2 years interval	11 [9,13]	1.00			11 [9,13]	1.00		
1st birth rank	16 [14, 19]	1.47	1.16	1.87	16 [14, 19]	1.48	1.16	1.88
2nd/3rd birth rank, less than or equal to 2 years interval	16 [10, 22]	1.49	0.99	2.23	17 [11, 23]	1.53	1.02	2.29
4th birth rank, more than 2 years interval	102 [93, 111]	9.91	8.05	12.20	98 [89, 107]	9.12	7.38	11.25
4th birth rank, less than or equal to 2 years interval	19 [10, 27]	1.79	1.09	2.92	20 [11, 29]	1.87	1.15	3.06
**Previous Death of Sibling**								
No	28 [26, 30]	1.00			28 [26, 30]	1.00		
Yes	37 [23, 51]	1.31	0.88	1.96	38 [23, 52]	1.36	0.90	2.04
Number of children born*								
1	35 [31, 39]	1.00			34 [34, 38]	1.00		
2	21 [18, 24]	0.57	0.47	0.69	21 [17, 24]	0.58	0.48	0.71
3	20 [17, 24]	0.58	0.46	0.73	20 [16, 24]	0.58	0.46	0.74
4+	25 [21, 29]	0.70	0.57	0.85	25 [21, 29]	0.73	0.59	0.89
**Number of children under-five years**								
1–2	33 [31, 36]	1.00			32 [30, 35]	1.00		
3+	20 [18, 23]	0.61	0.52	0.71	21 [18, 23]	0.63	0.53	0.74
**MEDIA FACTORS**								
Watches TV every week*								
Yes	25 [22, 27]	1.00			24 [22, 27]	1.00		
No	33 [30, 36]	1.36	1.18	1.58	33 [30, 36]	1.38	1.19	1.60
Listens to radio every week*								
Yes	31 [26, 36]	1.00			31 [26, 36]	1.00		
No	28 [26, 30]	0.90	0.76	1.08	27 [25, 30]	0.88	0.74	1.06
Read newspaper*								
Yes	19 [14, 23]	1.00			18 [14, 22]	1.00		
No	30 [28, 32]	1.61	1.27	2.05	30 [27, 32]	1.63	1.27	2.10
**ENVIRONMENTAL FACTOR**								
Type of cooking fuel*								
Solid fuel	18 [13, 23]	1.00			28 [16, 39]	1.00		
Non-solid fuel	31 [28, 33]	1.56	1.18	2.04	37 [33, 41]	1.34	0.82	2.18

*Rates did not add up because of missing values.

Note: 95% confidence intervals (CI) that include 1.00 indicate a non-significant result.

(a) Overall Bangladesh population

Multivariable analyses were performed with and without Rangpur division and showed that there was no substantial statistical difference between inclusion or removal of Rangpur division from the data sets. In this study, we provide interpretation of findings for all regions of Bangladesh (analyses with Rangpur division). In the multivariable analyses, the odds of stillbirth were significantly lower in educated mothers compared to those who had no schooling (Table 3). The risk of stillbirth was significantly higher among mothers from poorer households compared to those from rich households. Mothers with four or more children were significantly less likely to have a stillbirth compared to those who had one child. Mothers who did not read newspapers every week were significantly more likely to experience a stillbirth compared to those who read newspapers every week.

**Table 3.** Predictors of stillbirth: adjusted odds ratio (AOR) in Bangladesh, 2004–2014.

	With Rangpur (a)				Without Rangpur			
Characteristic	AOR	(95%CI)		P value	AOR	(95%CI)		P value
**Year of survey**								
2004	1.00				1.00			
2007	0.81	0.66	1.00	0.045	0.75	0.61	0.93	0.010
2011	0.54	0.44	0.66	<0.001	0.52	0.42	0.65	<0.001
2014	0.47	0.38	0.59	<0.001	0.41	0.32	0.52	<0.001
**Maternal highest level of education**								
No schooling	1.00				1.00			
Primary	0.66	0.55	0.80	<0.001	0.67	0.55	0.81	<0.001
Secondary or more	0.59	0.43	0.82	0.002	0.63	0.44	0.89	0.008
**Household Wealth Index**								
Rich	1.00				1.00			
Middle	1.30	1.01	1.66	0.040	1.51	1.14	2.01	0.004
Poor	1.47	1.13	1.90	0.004	1.62	1.21	2.16	0.001
**Number of children born**								
1	1.00				1.00			
2	0.56	0.46	0.69	<0.001	0.57	0.46	0.70	<0.001
3	0.49	0.39	0.63	<0.001	0.49	0.38	0.63	<0.001
4+	0.53	0.43	0.66	<0.001	0.51	0.40	0.65	<0.001
**Number of children under-five years**								
1–2	1.00				1.00			
3 or more	0.74	0.63	0.88	0.001	0.76	0.63	0.91	0.003
**Read newspaper**								
Yes	1.00				1.00			
No	1.34	1.02	1.76	0.037	1.38	1.03	1.86	0.033

Independent variables adjusted for community and socio-economic, child, media and environmental factor.

(a) Overall Bangladesh population

## Discussion

The study found that the rates of stillbirth were lower in 2014 compared to 2004. Stillbirth rates were higher in rural areas compared to urban areas in Bangladesh, and low maternal education, poor household, and having one child (primiparity) were significant predictors of stillbirth in Bangladesh. A further stratified analysis (with or without Rangpur division) showed no substantial statistical differences in the results.

The finding that stillbirth declined during the decade 2004–2014 is consistent with previous studies which reported lower rates of stillbirth in Bangladesh between 2009 and 2015 [11,12] and from 1990 to 2015 [1]. The reduction in the rates of stillbirth in Bangladesh has been attributed to a range of maternal and newborn interventions and socioeconomic policies. These include overall economic growth; improved education and social empowerment of women; increased health sector financing and investment; the scale-up of family planning services; and increased access to skilled birth attendants and expansion of the private health sector [22]. The marked improvement in child survival may also be due to the broader influence of programmatic commitments to the MDG’s between 1990 and 2015. Notably, the United Nations reported that Bangladesh was among the few countries worldwide to meet MDG-4 and MDG-5 (reduction of under-5 and maternal mortalities) [3]. While under-5 and maternal mortality rates are not direct measures of stillbirth rate, improvement in appropriate antenatal care, skilled births assistance and newborn care have been described as the core solutions to ending preventable stillbirth [22,23].

Although our study observed no association between maternal age and stillbirth, previous studies from developing countries such as Sudan [24] and Nigeria [25] and developed countries such as Australia [26] and the USA [27] have reported a higher risk of stillbirths in women aged over 35 years. The higher rate of stillbirths among older women may be due to increased risk of congenital anomalies associated with advanced maternal age. In contrast, hospital-based studies conducted in India [28] and Nigeria [29] reported an increased risk of stillbirths in mothers aged less than 20 years. This finding may reflect a lack of education, limited autonomy to make household decisions and poor health-seeking behaviours among teenage women, as reported in Nigeria [30] and India [31]. Nevertheless, a population-based study from Taiwan reported an increased risk of stillbirths in both older (>40 years) and younger mothers (<20 years) [32].

Consistent with previous studies conducted in developing countries, from rural Bangladesh to Uganda [10,11,33,34], this study showed that stillbirth rates were higher among mothers with no formal education compared to educated mothers. A study conducted in Norway indicated that stillbirth rates were higher in Norwegian women with fewer years of education, but not among Pakistani immigrant women in Norway [24]. In addition, our study found that mothers from poor households were more likely to experience stillbirths compared to those from rich households. A link between poverty and higher rates of stillbirth has been documented in developing countries [35,36], and a combination of no formal education associated with low-income family income may act as a major obstacle to timely and appropriate decision to seek early medical care in pregnant women. Our study provides supportive evidence that a lack of maternal education is associated with an increased risk of stillbirth in Bangladesh. This finding will assist public health campaigners advocating for targeted socio-educational initiatives to increase female education in Bangladesh.

In Bangladesh, the proportion of women who give birth at home with assistance from a traditional birth attendant (TBA) remains high [17], highlighting the poor uptake of appropriate perinatal health services such as antenatal care (ANC) and birth assistance from skilled health professionals. Antenatal care is an essential public health intervention and is recommended for all pregnant women worldwide by the WHO, based on evidence underpinning its importance in improving maternal and child health outcomes. However, in rural Bangladesh in particular, a range of factors have been linked with the persistent use of home birthing with TBA’s [37] including; traditional beliefs, poverty, religious fallacy, poor road networks, limited knowledge on the importance of healthcare services and a shortage of skilled health workers. Bangladesh would likely see further substantial improvements in child survival by implementing interventions that increase access to, and use of perinatal services, particularly among mothers in rural settings and other high-risk groups.

This study revealed that the risk of stillbirth was lower in mothers who had more than two children compared to those with one child, consistent with findings from previous studies, which indicated that stillbirth rates were higher among primiparous women [26,38]. In this setting this could be partly attributed to the young age of first-time mothers which is also a known risk for stillbirth, and lower use of health services and knowledge of the importance of timely and routine ANC.

The study findings have policy implications for public health experts, policy decision-makers, health administrators and developmental partners in Bangladesh. The Lancet Series [2,4,8] suggest a roadmap for ending preventable stillbirths. These include stronger independent accountability within countries, the establishment of stillbirth prevention strategic plans, empowerment of women and families, ensuring skilled birth attendance in health facilities, reduction in stigma associated with stillbirths and improvement in bereavement care. Achievement of SDG-3.2 (end preventable deaths of newborns and children under-five years of age by 2030) appears feasible in Bangladesh given the country’s MDGs achievement, however, targeted financial investment and strong political commitment are required. Furthermore, achievement of SDG-3.2 in Bangladesh would require collaborative efforts among various government and non-government agencies at both national and sub-national levels, as well as drawing experiences and capacities from the implementation of the MDGs agenda.

### Strengths and limitations

The following limitations should be considered when interpreting the study findings. First, the study used cross-sectional data, and a temporal association between exposure variables and the outcome cannot be determined. Second, the diagnosis of stillbirth was based on self-report, and this is a likely source of recall bias as respondents may incorrectly recall the gestational age they experienced a stillbirth. Third, data on other potential predictors of stillbirths (such as antepartum and intrapartum events, congenital anomalies or maternal drug use) as reported elsewhere [39] were not available. This latter information would have provided an additional contextual understanding of determinants of stillbirths in Bangladesh. Fourth, the study used pooled cross-sectional data, where population characteristics may differ over time. However, we adjusted for period and intra-cluster variability [40]. Additional information on the broader limitations of the DHS data utilisation has been described elsewhere [41].

Despite these limitations, the study has several specific strengths. First, selection bias is unlikely to affect the study findings, given the nationally representative sample and the high response rates that averaged 98%. Second, the BDHS used standardised questionnaires for data collection that provides population-based information on maternal and child health over time, allowing comparability across populations and time. Third, the data were collected by high-quality interviewers, which reduces the potential effect of interviewer bias. Fourth, this study provides country-wide evidence on predictors of stillbirths to health experts who can advocate for interventions to improve child survival and health at the national level in Bangladesh.

## Conclusion

Our analysis showed that rates of stillbirth were lower in 2014 compared to 2004 in Bangladesh, and risk factors for stillbirth were low maternal education, primiparity and poor household. These findings highlight the need for collaborative efforts to end poverty, ensure healthy lives for all, promote inclusive and equitable education, and empower women to improve child survival in Bangladesh. Drawing lessons from the implementation of MDGs would help accelerate progress towards achievement of ending preventable stillbirths in Bangladesh by 2030.

## Paper context

Globally, stillbirth is a significant public health issue, particularly in developing countries such as Bangladesh. We examined nationally representative data to identify potential predictors of stillbirths in Bangladesh over a ten-year period. Our study found that stillbirth rates were higher in rural areas, and no maternal education, poor household and primiparity were predictors of stillbirths in Bangladesh. There is need for more collaborative action to end preventable deaths and improve child survival in Bangladesh.
